# Spontaneous accessory renal artery aneurysm rupture as a first presentation of polyarteritis nodosa: a case report and review of literature

**DOI:** 10.1097/MS9.0000000000003331

**Published:** 2025-04-25

**Authors:** Raya N. Amro, Lana Maraqa, Amal A. Abo Jheasha, Essa Amro, Ruba Amro, Raghad H. M. Alwahsh, Saed Attawna

**Affiliations:** a Al-Quds University Faculty of Medicine, Medical Research Club, Jerusalem, Palestine; b Al-Ahli Hospital, Hebron, Palestine; c Al-Quds University Faculty of Medicine, Jerusalem, Palestine

**Keywords:** accessory renal artery rupture, case report, gangrenous bowel, polyarteritis nodosa, retroperitoneal hemorrhage

## Abstract

**Introduction:**

Polyarteritis nodosa (PAN) is a systemic necrotizing vasculitis primarily affecting medium-sized vessels. It has several clinical manifestations, including renal, gastrointestinal, cutaneous, neurologic, and general symptoms, but it is not associated with pulmonary manifestations. PAN mainly affects individuals aged 40–60 years, with a male predominance. Although the underlying cause of this disease remains unclear, several triggers can be associated with it such as hepatitis B virus. Diagnosis typically requires an organ biopsy or angiography revealing microaneurysms or stenotic lesions. The overall prognosis can improve with early diagnosis and administration of immunosuppressants. However, it remains a potentially life-threatening diagnosis with a mortality rate of 24.6% at 5 years for severe cases. We presented a rare case of PAN with severe renal and gastrointestinal involvement at presentation.

**Case presentation:**

A 21-year-old male presented with sudden severe right flank pain radiating to the back. He was noted to have reticular purple skin lesions on his abdomen and lower legs. Clinical and laboratory findings indicated that he had a hemorrhagic shock. A contrast-enhanced computed tomography (CT) scan of the abdomen and pelvis revealed a ruptured, partially thrombosed pseudoaneurysm of an accessory right renal artery with acute retroperitoneal hemorrhage, splenic infarcts, and two lower mesenteric artery aneurysms. Selective coil embolization of the ruptured artery was successfully conducted. Notably, the patient reports a 1-year history of intermittent abdominal pain, bilateral foot pain, and livedo reticularis, alongside left testicular pain managed with varicocelectomy. During the hospital stay, the patient developed progressive bilateral lower limb weakness that nerve conduction studies revealed it as mononeuritis multiplex. These combined findings were pointing toward PAN diagnosis. Therefore, the patient was started on pulse steroid and cyclophosphamide therapy. However, his abdominal pain worsened requiring surgical exploration with extensive bowel resection, after which plasmapheresis was commenced.

**Discussion:**

After our patient presented with life-threatening retroperitoneal bleeding, CT scan revealed that an accessory renal artery had ruptured, and also revealed the hidden cause of his chronic, recurrent, self-resolving attacks of severe abdominal pain, which were investigated multiple times by endoscopy without appropriate diagnosis. The final diagnosis of PAN was supported by the presence of livedo reticularis, testicular ischemia, chronic abdominal pain, and mononeuritis multiplex, fulfilling four diagnostic criteria of the American College of Rheumatology for PAN. Renal involvement in PAN can be in up to 75% of cases. However, rupture of accessory renal artery aneurysms is infrequent and it was the first presenting symptom of our patient. Similarly, GI complications are observed in 50% of patients, which can progress to life-threatening ischemia and gangrene, as seen in this case. Treatment involved corticosteroids and cyclophosphamide as an induction therapy based on the 2011 revised Five-Factor Score (FFS). The addition of plasma exchange therapy in this patient was due to the catastrophic complications of PAN. Eventually, the patient became clinically stable with an expected 5-year survival rate of 65.0% according to his FFS ≥2. Therefore, careful follow-up is necessary.

**Conclusion:**

Even though vascular aneurysms in PAN have a long history, they are more often linked to gradual development rather than to catastrophic events. Acute rupture resulting in hemorrhagic shock is rarely the initial sign of PAN. Rare reports of renal artery rupture in PAN highlight the significance of having a high level of clinical suspicion in young patients with unexplained vascular events. Early diagnosis and rapid management, including immunosuppressive therapy and plasmapheresis, are crucial in preventing severe outcomes. Despite a poor prognosis associated with severe disease features, careful management can stabilize the patient, although long-term follow-up remains essential.

## Introduction

Polyarteritis nodosa (PAN) is a disease characterized by systemic necrotizing vasculitis primarily affecting medium-sized vessels. While small-sized vessels, such as arterioles, capillaries, and venules, are usually spared^[^1^]^. It has a variety of clinical manifestations, including general symptoms, and neurologic, cutaneous, renal, and gastrointestinal manifestations. However, pulmonary manifestations and glomerulonephritis are not associated with the disease^[^2^]^. An organ biopsy or an angiography is generally required to confirm a diagnosis. Mixed-cell inflammatory infiltrates and fibrinoid necrosis are typical histologic findings, with the absence of granulomas and giant cells. Angiography findings may reveal multiple micro-aneurysms or stenotic lesions in visceral imaging^[^3^]^.

This disease can impact individuals of various ethnic backgrounds, predominantly presenting in those aged 40–60 years, and, in contrast to most other forms of vasculitis, exhibits a male predominance (male-to-female ratio of 1.5:1)^[^4^]^. It is estimated that there are 2–31 cases of PAN per million inhabitants in Europe, with a wide variation in prevalence between countries^[^5^]^.

The overall prognosis can improve with early diagnosis and administration of immunosuppressants. However, it remains a potentially life-threatening diagnosis with a mortality rate of 24.6% at 5 years for severe cases^[^6^]^.

We present a case of severe PAN with rare renal and gastrointestinal life-threatening presentation, aiming to deepen the understanding of this disease, aid in clinical diagnosis and treatment, and improve patient prognosis.

This case report has been prepared and reported in line with the CARE criteria^[^7^]^.

## Case presentation

**Figure 1.**
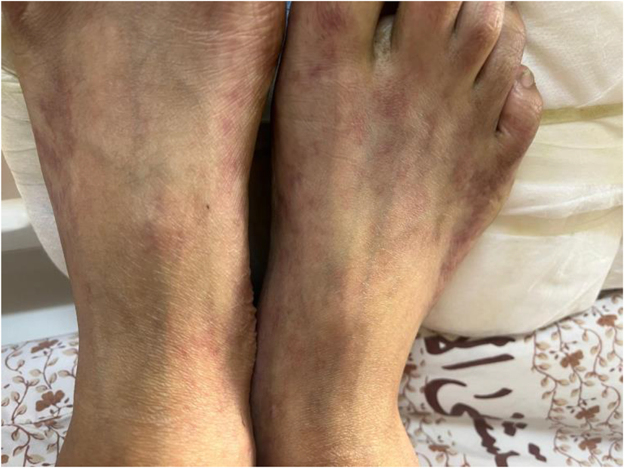
Reticular and purple skin lesions over lower limbs.

A 21-year-old male presented to our hospital with complaints of right flank pain radiating to his back, which was unresponsive to pain relief medications. He had developed a rash characterized by reticular and purple skin lesions, primarily on his abdomen and lower legs (Fig. 1). The patient also reported experiencing chills and significant confusion. Notably, he had been suffering from bilateral foot pain for over a year and had abdominal pain and discomfort for the past year. He also suffered from left testicular pain during the last year, managed as varicocele, and underwent varicocelectomy. Initially, he reported intermittent abdominal pain of an unclear origin despite multiple consultations with various physicians.

**HIGHLIGHTS**

- Polyarteritis nodosa (PAN) is a disease characterized by systemic necrotizing vasculitis primarily affecting medium-sized vessels.
- PAN can impact individuals of various ethnic backgrounds, predominantly presenting in those aged 40–60 years.
- The overall prognosis can improve with early diagnosis and administration of immunosuppressants.

On the day of his admission, he sought medical attention due to worsening pain in the right flank. Clinical evaluation indicated tachypnea and hypotension; routine urinalysis and liver function tests appeared normal. Laboratory results showed a white blood cell count of 21, a hemoglobin level of 7.5 g/dL (despite his family indicating it had been 11 g/dL previously), a mean corpuscular volume of 75 fL, a reticulocyte count of 1.65%, a C-reactive protein level of 104.06 mg/dL, a creatinine (Cr) level of 1.3 mg/dL, and a random blood glucose measurement of 530 mg/dL. Arterial blood gas analysis indicated a pH of 7.2, PaCO_2_ of 28.9, HCO_3_ of 12.3, and an anion gap of 16, consistent with anion gap metabolic acidosis.

**Figure 2.**
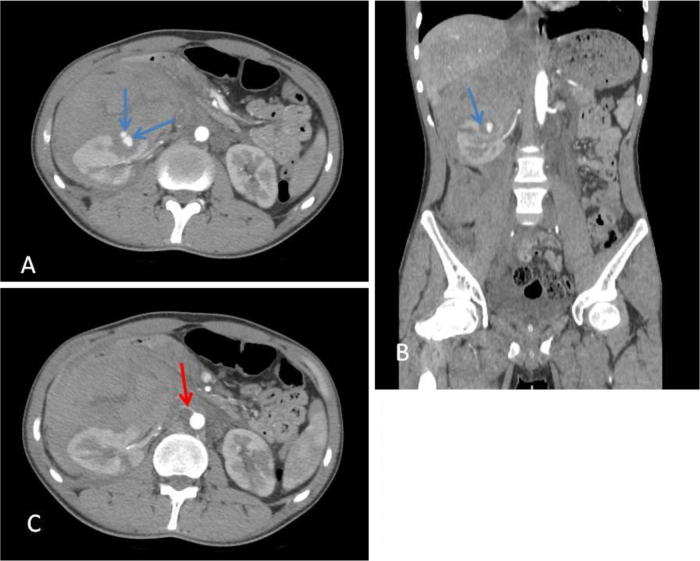
Axial (A) and coronal (B) views of post-contrast-enhanced computed tomography scan show partially thrombosed pseudoaneurysm (blue arrows) and accessory right renal artery (red arrow).

**Figure 3.**
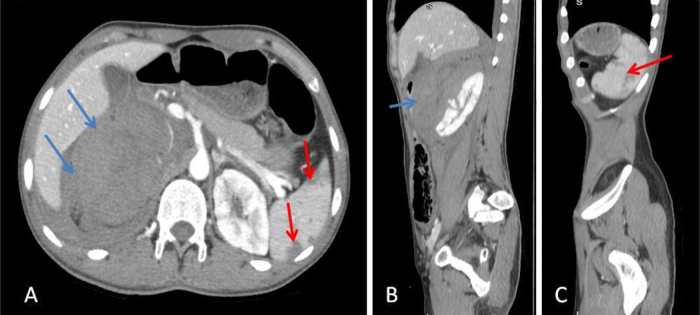
Axial (A), right parasagittal (B), and left parasagittal (C) views of post-contrast-enhanced computed tomography scan show retroperitoneal hematoma with contrast extravasations (blue arrows) and multiple splenic infarctions (red arrows).

**Figure 4.**
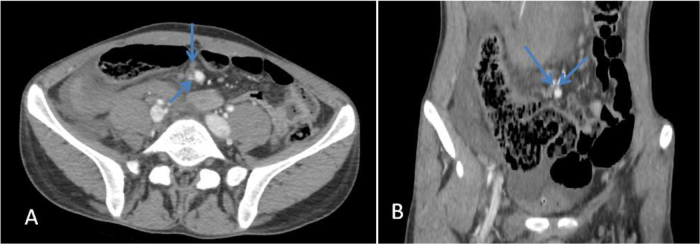
Axial (A) and coronal (B) views of post contrast-enhanced computed tomography scan show partially thrombosed superior mesenteric artery aneurysm (SMA) (blue arrows).

A computed tomography (CT) scan of the abdomen and pelvis, performed with intravenous contrast, identified a ruptured, partially thrombosed pseudoaneurysm located in the right central renal pole, likely originating from an accessory artery in the right lower pole (Fig. 2). This finding was associated with acute retroperitoneal bleeding and active hemorrhage, along with two aneurysms measuring approximately 10 mm each from the lower SMA branches (one appearing partially thrombosed) in the mid-anterior abdominal cavity (Fig. 4), alongside several small splenic infarcts (Fig. 3). In response, the patient was immediately placed on aggressive IV fluids, antibiotics, and transfusions for early hemorrhagic shock. He subsequently underwent embolization of the right accessory renal artery utilizing a Nester micro-coil (Fig. 4).

We engaged the surgical team to assess the patient’s vitality, leading to the decision to continue observation without immediate surgical intervention. Throughout the hospital stay, the patient experienced escalating pain in the lower limbs, ultimately resulting in weakness and an inability to move. In response, we contacted the neurology and rheumatology departments for further evaluation. Nerve conduction studies of the lower extremities revealed relatively low compound muscle action potentials and signs of conduction blockage in both tibias at the knee, accompanied by absent F waves and reduced sensory potentials, indicating a decrease in nerve conduction velocity. These findings align with a diagnosis of polymyositis. Subsequently, an magnetic resonance imaging of both thighs with venous contrast was conducted to exclude myositis, which showed that the muscles appeared normal and displayed no abnormal signals.

From a rheumatological perspective, the patient’s symptoms pointed toward PAN, warranting further investigations. Tests for hepatitis B and C, antinuclear antibodies, antineutrophil antibodies, and complement levels C3 and C4 all returned negative results. Additionally, a transesophageal echocardiogram and a testicular ultrasound indicated notable dilation of both the upper and lower poles of the left scrotum, measuring up to 3.2 mm, suggesting spontaneous reflux in a supine position, consistent with severe varicocele. The examination revealed that both testes displayed heterogeneous echogenicity, more pronounced on the left side, but no signs of focal disease or orchitis were observed. Echocardiography was done and came back to normal results. The left inguinal incision was opened layer by layer, revealing the left spermatic cord, which was carefully identified and preserved. The vas deferens and the ilioinguinal nerve were also located and preserved during the procedure. The spermatic vein was ligated, and hemostasis was successfully secured.

**Figure 5.**
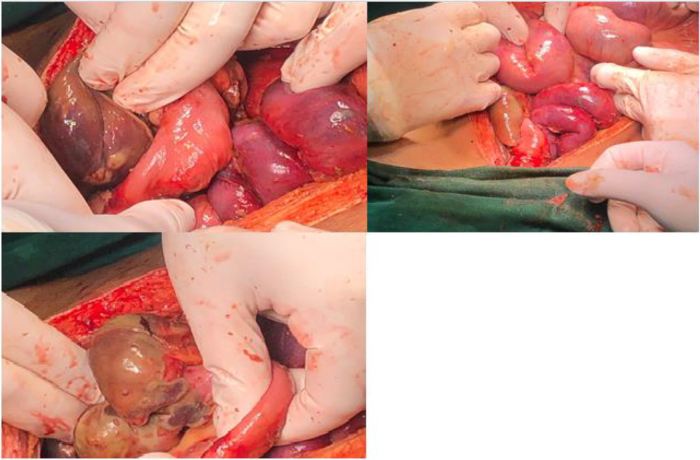
Ischemic colitis and a gangrenous appendix.

Due to a suspected rheumatological issue, the patient was started on pulse steroid, cyclophosphamide therapy, and antibiotics. During hospitalization, the patient began to experience abdominal pain and bloody diarrhea. A colonoscopy was performed, revealing multiple anorectal ulcers with bleeding, raising concerns of possible bowel ischemia. Conservative treatment was attempted, leading to a temporary improvement in the patient’s condition for a few days before a relapse occurred. Consequently, the patient underwent exploratory laparotomy to address ischemic colitis and a gangrenous appendix (Fig. 5), resulting in the resection of 90 cm of unhealthy intestine from the ligament of Treitz to the transverse colon, as well as ileal perforation and retroperitoneal hematoma. The surgical procedure included the resection of the gangrenous bowel through an extended hemicolectomy and right iliac approach. The transverse colon was closed using a stapler, while the small bowel was manually closed before being returned to the abdominal cavity, which was kept open by suction. Following this, a second open surgery was performed under general anesthesia. A colonoscopy conducted by the GI team showed an acceptable condition of the colon, with a thorough examination of all bowels and organs indicating that the small bowel displayed good color and peristalsis, with only mild edema observed, and the large bowel also appeared to be in good condition. Minor patches of grey color were noted at the rectum, but overall it seems viable. The abdomen was cleaned, and hemostasis was achieved. A release of skin was performed through component separation to alleviate tension. The transverse colon was exteriorized via the right-sided opening of the colostomy, while the jejunum was exteriorized through the left opening. Pelvic and subcutaneous drains were inserted and secured, followed by skin closure after thorough cleaning. The colostomy and ileostomy were affixed to the skin, demonstrating good blood supply and viability, with continuous dressing applied. The patient was stable both clinically and in laboratory results. plasmapheresis was initiated to prevent further relapse.

## Discussion

PAN is a systemic vasculitis distinguished by necrotizing inflammatory lesions impacting medium-sized and small muscle arteries, particularly around artery bifurcations. These lesions lead to the production of microaneurysms, aneurysmal rupture accompanied by hemorrhage, thrombosis, and ultimately, organ ischemia or infarction^[^1^]^.

PAN is mainly a primary disease, but it is also linked to several conditions, including viral infections, especially hepatitis B virus (HBV); hematological malignancies, such as hairy T cell leukemia, or myelodysplastic syndrome; familial Mediterranean fever (FMF); and the use of certain drugs, such as minocycline^[^8^]^.

Our case presents a 21-year-old male patient from Palestine, who is free of HBV and FMF. He was diagnosed with PAN after multiple steps were taken during the admission leading to this diagnosis. The presentation of PAN in young males is exceedingly rare, making this case particularly noteworthy, as similar cases with severe presentations occurred in much older patients.

American College of Rheumatology (ACR) has classified PAN based on 10 criteria, which include weight loss ≥4 kg, livedo reticularis, testicular pain or tenderness, myalgias, mononeuropathy or polyneuropathy, diastolic blood pressure >90 mm Hg, elevated blood urea nitrogen or serum creatinine levels, presence of hepatitis B reactants in serum, arteriographic abnormality, and presence of granulocyte or mixed leukocyte infiltrate in an arterial wall on biopsy. The presence of 3 or more of these 10 criteria was associated with a sensitivity of 82.2% and specificity of 86.6%^[^9^]^.

After our patient presented with life-threatening retroperitoneal bleeding, a CT scan revealed that an accessory renal artery had ruptured, and also revealed the hidden cause of his chronic, recurrent, self-resolving attacks of severe abdominal pain, which were investigated multiple times by endoscopy without appropriate diagnosis. Additionally, he had livedo-reticularis for 2 years without seeking medical advice for a proper diagnosis because of his low socioeconomic status. Furthermore, he complained of testicular pain and underwent a varicocelectomy, even though an ultrasound showed ischemic changes to his testes. As a result, the combination of these missed earlier signs and symptoms fulfills four of the ACR diagnostic criteria, and the vascular events are thus attributed to PAN.

As our patient has had bilateral lower limb pain since admission, several diagnostic approaches have been taken to find out the underlying cause of that pain. Finally, it turned out that this pain was a manifestation of mononeuritis multiplex, which is a common neurological complication in PAN that occurs in approximately 60% of patients. It results from damage to the vasa nervosum due to the inflammation from the underlying vasculitis and thrombosis. Therefore, the infarction of the corresponding nerve manifests as pain^[^10,11^]^.

The diagnosis of mononeuritis multiplex was made based on nerve conduction studies and electromyography of the lower extremities.

PAN often involves the renal artery and its branches in up to 75% of cases, causing stenosis or aneurysm, affecting the renal and interlobar arteries in particular, and less commonly the smaller arcuate and interlobular arteries. There are several manifestations of kidney involvement, including hypertension, hematuria, proteinuria, renal infarction, and hemorrhage^[^12^]^.

Severe, life-threatening complications such as ruptured aneurysm and spontaneous perirenal hemorrhage, which require embolization or nephrectomy, are infrequent, but increase with clinical severity of the disease, as reported in similar cases.

To our knowledge, four other cases of severe renal PAN presentation have been reported in the literature^[^13^−^16^]^ (Table 1).

**Table 1:** Summary table of case reports of severe PAN with spontaneous renal aneurysmal rupture

Case reference	Age/gender	Main presentation	Diagnosis	Intervention	Outcome
Smith *et al*, China, 2023^[^13^]^	Male, 58	Renal aneurysm rupture, intestinal perforation	CT angiography	Corticosteroids, cyclophosphamide	Stable state
Lee *et al*, Japan, 2020^[^14^]^	Male, 67	Perirenal hematoma	CT angiography	Selective coil embolization, corticosteroids, cyclophosphamide	Stable state
Ullah *et al*, USA, 2019^[^15^]^	Male, 67	Retroperitoneal hemorrhage	CT angiography	Embolization and corticosteroids	Stable state
Gomez *et al*, Australia, 2017^[^16^]^	Male, 85	Recurrent spontaneous renal hemorrhage	CT angiography	Selective coil embolization, Corticosteroids, cyclophosphamide	Stable state

CT, computed tomography.

In our case of severe PAN, the patient presented with flank pain and features of hemorrhagic shock. The diagnosis of ruptured partially thrombosed pseudoaneurysm arising from an accessory artery active bleeding was revealed by a CT scan. Rapid intervention to stabilize the patient was required and nester micro coil embolization to the ruptured micro-aneurysm in the accessory artery was successfully conducted without complications.

The involvement of an accessory renal artery (ARA) has special considerations. The overall prevalence of ARAs is estimated to be 21.10%, and it has been linked to resistant hypertension due to its suggested role in activating the renin-angiotensin-aldosterone system^[^17^]^.

This is thought to be increasing the risk of aneurysm formation in the artery leading to its rupture making it unique to our case.

Gastrointestinal signs of PAN are prevalent, affecting up to 50% of patients, and are associated with a high mortality rate. Mild cases may present with abdominal pain, generally appearing as postprandial pain or abdominal cramps. Severe vascular inflammation of the mesenteric arteries can lead to intestinal ischemia, perforation, and bleeding, Additionally, 10% of people with PAN have experienced gastrointestinal bleeding or required abdominal surgery due to gastrointestinal complications^[^13^]^.

Based on that, this highly explains our patient’s history of the previous vague chronic intermittent abdominal pain, for which he underwent several endoscopic evaluations and was presumably diagnosed as atypical gastritis with mild symptomatic improvement on the prescribed proton pump inhibitors.

Later after the patient’s stabilization, he developed a relapsing course of tense abdomen and bloody diarrhea. This is explained in light of his PAN diagnosis and vasculitis-induced intraluminal thrombosis of the two inferior SMA branches. The need to do surgical exploration was determined and revealed gangrenous bowels, resulting in a right hemicolectomy.

The treatment of PAN is highly dependent on the severity of pain, with considering the glucocorticoid as the mainstay of treatment, adding further drugs is possible based on the severity and the response of the disease^[^9,18^]^.

Four factors indicate the prognosis of PAN including age (older than 65 years), renal insufficiency (serum creatinine >1.7 mg/dL), cardiac insufficiency, and severe gastrointestinal involvement according to the 2011 revised Five-Factor Score (FFS)^[^6^]^. As it is recommended for cases of PAN with an FFS ≥1 cyclophosphamide can be used as induction therapy.

FFS in our case was 2 based on severe renal and gastrointestinal involvement. Therefore, he was started on pulse steroid and cyclophosphamide therapy. Temporary improvement in the patient’s condition was achieved for a few days before a relapse occurred, after which five plasmapheresis sessions were done, and no further deterioration or relapse in his clinical or laboratory evaluations, which was considered as being clinically stable.

The addition of plasma exchange therapy in this patient was due to the catastrophic complications of PAN including the resultant organ ischemia that occurred a few days after the admission. Thus, although our patient is HBV negative, the addition of Plasmapheresis here was according to the rheumatologist’s clinical expertise of its use as an additive treatment to further control the disease and prevent relapse^[^9,19^]^.

The 5-year survival rate in PAN with an FFS ≥2 is 65.0%, careful follow-up is necessary^[^6^]^.

In conclusion, even though vascular aneurysms in PAN have a long history, they are more often linked to gradual development than to catastrophic events. Acute rupture resulting in hemorrhagic shock is rarely the initial sign of PAN, even though renal artery aneurysms are among the most often reported conditions. Rare reports of renal artery rupture in PAN highlight the significance of having a high level of clinical suspicion in young patients with unexplained vascular events.

## Data Availability

Not available.

## Methods

The work has been reported in line with the SCARE 2023 criteria^[^7^]^.
